# An Anomalous Case of Replantation

**Published:** 1879-02

**Authors:** A. W. Harlan

**Affiliations:** Chicago, Ill.


### AN ANOMALOUS CASE OF REPLANTATION.

BY A. W. HARLAN, CHICAGO, ILL.

EDITOR OF REGISTER.—DEAR SIR:—A gentleman in the endeavor to open the apex of the root of an upper left lateral incisor tooth, with a fine needle, was so unfortunate as to break off the needle in his tooth, after it had passed through the foramen a distance exceeding an eighth of an inch. This accident happened on Sunday morning, at four a. m , the 26th of May, 1878. He was sitting on my office steps when I arrived there on Monday at half past eight a. m.

I worked an hour or more trying to dislodge the remnant of the needle, but my attempt to do so was futile. Not having more time at my disposal, he was dismissed until half past four p. m., having previously depleted the gum. I did not know at the time that the needle had passed through the end of the root, and as he had not preserved the other portion of the needle, I was unable to determine that point. The root, as I found afterwards, was a trifle more than three-fourths of an inch in length, and that portion of the needle which had not passed through the apex, was not longer than the thirty-secondth part of an inch--rendering it impossibl- to dislodge it in the ordinary manner. At the appointed time the gentleman returned in great agony. After a few minutes I extracted the tooth and found what has been related above. Here was a case for replantation, as the gentleman had only seven front teeth, and two molars on opposite sides, he readily agreed to my proposal to replace the tooth, which was done in somewhat less than an hour from the time it was extracted. The hour being late, I filled the crown with gutta percha, and the root with gold. Previous to the replacement of the tooth I held it in tepid water for a few moments, and also caused a free flow of blood from the socket, ligating it to the adjourning teeth, I bathed the gum daily two or three times, with tincture of arnica only. By the following Monday the tooth was firm in its socket. On the last day of July

I filled the crown of the tooth with gold, restoring one-fourth of it—using the mallet to consolidate the filling. There was no pain or soreness. The tooth is still in good condition—Jan. 29, 1879.

